# Gain control network conditions: the role of the inhibition

**DOI:** 10.1186/1471-2202-14-S1-P176

**Published:** 2013-07-08

**Authors:** Eduardo Serrano, Thomas Nowoty, Rafael Levi, Brian H Smith, Ramón Huerta

**Affiliations:** 1 GNB, Department of Computer Science, Universidad Autónoma de Madrid, Madrid, Spain; 2 CCNR, Informatics, University of Sussex, Brighton, UK; 3 Department of Neurobiology and Behavior, University of California, Irvine, CA, USA; 4 School of Life Sciences, Arizona State University, Tempe, AZ, USA; 5 BioCircuits Institute, University of California San Diego, La Jolla, CA, USA

## 

During neural information processing, solving decision making problems or performing pattern recognition in any sensory modality, the brain can recognize objects as the same thing regardless of the stimulus intensity. The regulation of the internal representation under various levels of extrinsic stimulation is called gain control [1].

However, the precise mechanisms for achieving effective gain control in the brain are unknown. Based on our understanding of the existence and strength of connections in the insect olfactory system, we analyze whether conditions exist that would lead to controlled gain. The question we want to address is to determine whether is possible to design stable neural circuits made of excitatory and inhibitory neurons capable of controlling the internal representation of the external stimulus using only the network properties. Using a mean field approximation for the network activity, we show that there is a precise relationship of network parameters that can account for stable internal representation regardless of the external stimulus. Our findings conclude that the most important network parameters are the connections from the inhibitory population to the rest. This is consistent with experimental findings [2]. We also show that the connections from the excitatory population to the inhibitory one do not play an important role in gain control, suggesting that they can be freed for encoding purposes without damaging the operating response of the network to increasing levels of stimulation. Finally, we confirm that the gain control conditions derived from the mean field approximation are valid in simulations of firing rate models and Hodgkin-Huxley conductance based models.

**Figure 1:**
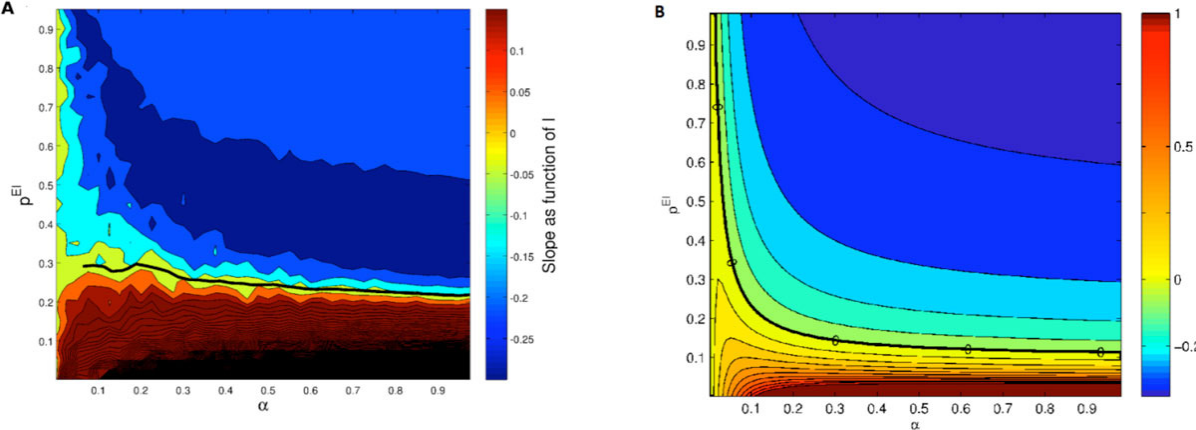
**Contour plot of slopes of response curves in numerical experiments using rate model neurons (A) and mean field equations (B)**. We plot the derivative of change in the PN activity with respect to the external stimulus as a function of α which is the fraction of excitatory neurons that receive input, and p^EI^, which is the probability of having a connection from an inhibitory neuron to an excitatory one. Perfect gain control corresponds to 0 slope and is represented by the thick black solid line in both panels.
